# Oxygen-releasing seed coating enhances yield and resource use efficiency in direct-seeded rice

**DOI:** 10.3389/fpls.2026.1746831

**Published:** 2026-01-27

**Authors:** Yuanqing Shi, Huilai Yin, Yuemei Zhu, Ruhongji Liu, Qiqi Chen, Hongkun Xie, Binbin Liu, Qingyue Cheng, Chuanhai Shu, Ning Liu, Jun Ma, Yongjian Sun, Na Li, Zhiyuan Yang

**Affiliations:** 1 Rice Research Institute, Sichuan Agricultural University, Chengdu, China; 2 Pilot-Scale R&D Platform of Sichuan Province for New Rice Varieties and Technologies, Meishan, China; 3 Crop Ecophysiology and Cultivation Key Laboratory of Sichuan Province, Chengdu, China

**Keywords:** direct-seeded rice, economic benefits, energy use efficiency, oxygen-releasing seed coating agent, yield

## Abstract

To address seed decay in direct-seeded rice caused by waterlogging resulting from inadequate field leveling, this study conducted split-split-plot field experiments in Chongzhou City, Sichuan Province (103°38’31’’–103°39’22’’ E, 30°33’16’’–30°33’54’’ N). Specifically, two hybrid rice varieties previously identified as flood-resistant (V1: Jinyou 1319) and flood-sensitive (V2: Jingliangyou 1377) were assigned to the main plots, wet direct seeding (P1) and water direct seeding (P2) were compared in the subplots, and the coating (C1) and no-coating (C2) treatments were applied to the sub-subplots. In the coating treatment with water direct seeding, the seedling percentage of V1 and V2 increased by 25.58% and 78.54%, respectively, the number of effective panicles increased by 4.69% and 12.95%, respectively, and the seed setting rate improved by 15.05% and 16.64%, respectively. This synergy boosted the yields of the two varieties by 23.15% and 31.77%. In particular, the yield of V2 with water direct seeding with coating matched that under wet direct seeding without coating. With little difference in total energy consumption (≤ 1.88%), the sensitive variety with water direct seeding and coating saved irrigation water and labor inputs by 13% and 17%, respectively, in the demonstration area (calculated based on the input of the demonstration area). With water direct seeding, the stable oxygen supply from the coating improved the seed germination rate and seedling growth vitality, especially for the sensitive variety. Thus, the oxygen-releasing coating achieved yield increases, resource conservation, and efficiency enhancement synergistically, providing a valuable solution for the development of direct-seeded rice in China’s hilly regions.

## 1 Introduction

As a staple food crop feeding half of the world’s population, the stable production of rice is directly tied to global food security. China, the largest rice producer worldwide, accounts for 28.5% of global rice production (Fukagawa and Ziska, 2019). However, the traditional rice transplanting pattern is facing serious challenges, such as high water consumption [65% of total agricultural water consumption (Li et al., 2020)], continuous increase in labor costs [annual average 12.3% (Lu et al., 2018)], and< 30% mechanization rates in small-scale plots (< 0.3 hm^2^) (Yang et al., 2023). Direct-seeded rice, i.e., sowing seeds directly in the field, eliminates seedling raising and transplanting, thereby significantly saving approximately 30% in water consumption and 50% in labor input (Nirmala et al., 2021). The advantages of direct-seeded rice have been demonstrated in various studies worldwide. In India and Pakistan, direct-seeded rice has achieved higher yields at lower production costs (Ishfaq et al., 2020; Mishra et al., 2017), and tests in China’s Yangtze River Basin have also demonstrated its significant effectiveness in water conservation compared to the traditional transplanting pattern (Tao et al., 2016). Overall, the water-conservation, cost, and efficiency advantages have made this modern planting pattern an important measure to address resource and labor constraints.

Currently, direct-seeded rice is implemented using the dry direct seeding, water/water direct seeding, and wet direct seeding methods. With dry direct seeding, seeds are sown into dry soil, which reduces irrigation water consumption by 30% to 40% compared to rice transplanting (Cabangon et al., 2002). Nevertheless, its prolonged bare-soil period creates vulnerability to weed invasion, often leading to severe weed infestations (Wu et al., 2024). Meanwhile, dry direct seeding requires sowing seeds deep to retain water, which increases soil resistance for seed emergence and reduces the germination rate and seedling percentage (Chamara et al., 2018). With water direct seeding, seeds are sown into the soil with a static water layer, which inhibits weed germination (Zhang et al., 2025). However, the water layer also creates an oxygen-deficient environment, hindering rice seed germination and seedling establishment (Hu et al., 2022). Wet direct seeding, i.e., sowing seeds into saturated soil, establishes a slurry environment that reduces temperature fluctuations, thereby promoting seed germination and root development while reducing adversity stress in the seedling stage (Farooq et al., 2011). About 35% of China’s rice fields are distributed in hilly regions with a slope of > 5°, where large farming machinery struggles to operate, while small machinery performs poorly in field leveling. As a result, the fields are often uneven with potholes, leading to uneven water distribution for direct-seeded rice. Therefore, the heavy weed infestation and reduced seedling percentage caused by uneven fields remain core bottlenecks hindering the development of direct-seeded rice despite effective reductions in labor and water input.

Meanwhile, good field leveling cannot be guaranteed under actual conditions (Pan et al., 2017). Because of its greater adaptability to various field conditions, water direct seeding is often considered more promising for practical applications than wet direct seeding, which requires fine water management (Wang et al., 2025). Nonetheless, a key bottleneck of water direct seeding is the difficult seed germination and seedling establishment, as it leaves rice seeds in an oxygen-deficient environment for a prolonged period (Farooq et al., 2011). To address this obstacle, our research team focused on seed coating innovations and developed a proprietary oxygen-releasing coating agent. The coating agent leverages the oxygen-releasing properties of calcium peroxide (CaO_2_) upon dissolution in water to increase the oxygen concentration around the seed and alleviate hypoxia stress (Mei et al., 2017). Meanwhile, it integrates supplementary regulators, such as uniconazol, to enhance root respiration and further reduce hypoxia damage (Wang et al., 2022). In the meantime, the Ca(OH)_2_ produced during calcium superoxide activation can slightly increase the microenvironment pH to weak alkalinity, and the activation products can suppress pathogenic microbes, thereby promoting germination through improved oxygen, physicochemical, and microbial conditions (Więckol-Ryk et al., 2020). Compared with traditional coating agents that mainly serve fungicidal and nutritional functions, the oxygen-releasing coating agent can effectively alleviate the oxygen-deficient environment of water direct seeding. In theory, it can significantly improve seed germination rate and seedling percentage, making it a feasible technical solution for promoting water direct seeding in the uneven fields of hilly regions.

Admittedly, the performance of this oxygen-releasing coating agent in actual applications is still unclear. While researchers have preliminarily proven the potential of existing coating agents to improve seed germination, numerous gaps remain in global-scale research and testing, which limit the technology transfer from the laboratory to large-scale applications. For one thing, the existing research findings are derived from controllable test environments, and systematic evaluation and verification in actual field environments with abiotic stresses related to water and temperature are lacking. For another, the whole-life-cycle energy input-output and cost-benefit analyses for this technology remain too scarce to provide targeted decision-making support to practical production (Pedrini et al., 2017). To this end, this study conducts field tests to systematically evaluate its application value. Specifically, a four-dimensional evaluation system incorporating seedling percentage, yield, energy, and economy indices is constructed, and an experiment design coupling variety, coating, and direct seeding method is implemented in a typical hilly rice-planting region (Chongzhou, Sichuan, with a gradient of 4.2° to 8.7°). The focus of analysis in this study is (1) the net energy balance between the energy-saving benefits from irrigation and the input incurred by the coating, and (2) the marginal cost yields for small farmer household applications. With optimized energy and resource efficiency, this coating could increase rice yield of water direct seeding to the level of wet direct seeding, providing a sustainable solution for rice production in the hilly regions, which account for 35% of China’s rice-planting regions.

## 2 Materials and methods

### 2.1 Test materials and location

This study combined field tests with demonstration area surveys. The field tests were conducted to obtain seedling condition and yield data, while demonstration area surveys were performed to collect economic data, such as production material and labor inputs. Two hybrid rice varieties with significant differences in flood sensitivity, namely, Jinyou 1319 and Jingliangyou 1377, were selected for the experiments. Specifically, varieties were screened under continuous flooding (a 3 to 5 cm water layer) for 15 days, and the germination rate of Jinyou 1319 was significantly higher than that of Jingliangyou 1377 by an average of 9.46 percentage points. Under wet direct seeding (no water stress) conditions, however, the germination rates of the two varieties showed no significant difference. Therefore, Jinyou 1319 was defined as a flood-resistant variety, whereas Jingliangyou 1377 was a flood-sensitive variety. The required seeds were selected and disinfected by soaking in a 0.6% sodium hypochlorite solution for 15 min. After rinsing 3 times with ultra-pure water, the seeds were soaked in ultra-pure water at 25 °5 for 48 h and removed from the container for later use. The coating agent used in this study was a patented product independently developed by our research team. It was prepared using calcium peroxide (18%), Thiram (2%), sodium naphthalene sulfonate formaldehyde (1%), uniconazole (0.1%), eosin (0.15%), gum arabic (6%), talc (32%), and kaolin (30%). Briefly, the dry powders like Thiram and sodium naphthalene sulfonate formaldehyde were mixed first, and calcium peroxide was mixed into the powders last. The gum arabic was dissolved in ultra-pure water to produce an adhesive, which was mixed into the mixed powders, yielding the coating agent. The treated seeds were poured into a seed dresser to evenly coat the agent onto the seed surface, achieving a coating thickness of 2 ± 0.3 mm. Following low-temperature drying, the coated seeds were stored for later use.

**Figure 1:**
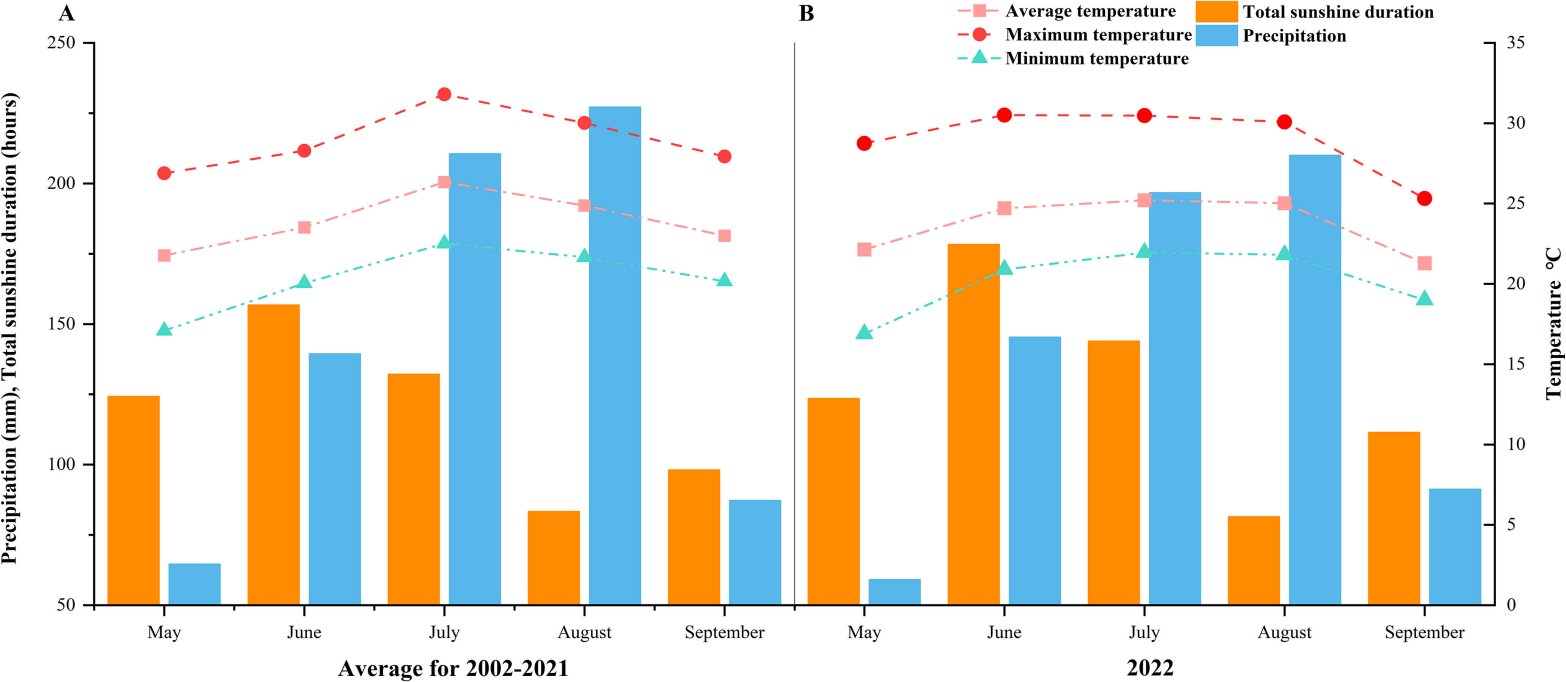
**(A)** Rice growth season meteorological data in Chongzhou (2002-2021); **(B)** Rice growth season meteorological data in Chongzhou (2022).

The tests were conducted in Chongzhou City, Sichuan Province (103°38’31’’-103°39’22’’ E, 30°33’16’’-30°33’54’’ N) in 2022, and the test site was located in the middle of the demonstration area. Sowing was performed on May 18. The arable layer soil (0 to 20 cm) at the test site was sandy loam with an organic matter content of 15.28 g·kg^−1^ and a total nitrogen content of 1.52 g·kg^−1^. The available nitrogen, available potassium, and available phosphorus were 84.23 mg·kg^−1^, 112.84 mg·kg^−1^, and 20.42 mg·kg^−1^, respectively. Regarding meteorological data, the 2012–2021 average data (**Figure 1**) were from the Sichuan Provincial Meteorological Service, and the 2022 data were from the small meteorological station erected in the test field (Licheng Automation Equipment Co., Ltd., LC-DZZ1-2).

### 2.2 Test design

The field tests adopted a three-factor split-split-plot design, with the two hybrid rice varieties (Jinyou 1319, V1; Jingliangyou 1377, V2) as the main plot factors. Two direct seeding methods, wet direct seeding (P1) and water direct seeding (P2), were assigned to the subplots. Two seed treatments, oxygen-releasing seed coating (C1) and no-coating (C2), were assigned to the sub-subplots. Thus, a total of 8 treatments were designed. With each treatment triplicated, a total of 24 plots were established, each 50 m^2^ (10 m × 5 m). Each factor level was randomly assigned to the plots to control for soil differences and micro-area environmental variability. The main plots were arranged randomly, and the subplots and sub-subplots were independently randomized within their respective upper-level plots.

Field water and agronomic management were strictly regulated. At 7 days before sowing, the field was subjected to deep plowing with standing water. Following a settling period, a dense, compacted layer was formed to reduce permeability, which maintained the water table. One day before sowing, the water layer was drained to 3 cm, and laser-assisted land leveling was performed (field height difference ≤ 3 cm) after applying the base fertilizer. Doing so unified the field surface conditions and water depths, thereby reducing the non-target differences between treatments. For wet direct seeding, a 3 cm water layer was maintained for 12 h after leveling, and the field was drained until no visible water on the field surface before hill seeding. Then, cycled wetting management was applied according to the soil water potential (−10 kPa as a threshold). For water direct seeding, the 3 cm water layer was maintained for 12 days, during which underwater seeding was performed. The water layer was drained after 12 days, and water management thereafter was the same as that under wet direct seeding. At 30 days following seeding, all treatments were subjected to unified water and fertilizer management, namely, shallow water at the tillering stage, field drying at the nonproductive tillering stage, and alternate irrigation at the booting and filling stages. The total amount of fertilization was 150 kg·hm^2^ of nitrogen (base, tillering, and panicle fertilizer ratio = 3:3:4), 75 kg·hm^2^ of P_2_O_5_ (all applied as base fertilizer), and 150 kg·hm^2^ of K_2_O (half applied as base fertilizer, half as tillering fertilizer). During seeding, the row spacing was 25 cm, the hill spacing was 15 cm, and 5 seeds were sown per hill.

### 2.3 Test items and methods

#### 2.3.1 Germination and seedling establishment survey

Following sowing, 5 sites were selected in each plot using the five-point sampling method, each including 3 rows × 10 hills/row = 30 hills, totaling 150 hills per plot for the germination rate and seeding percentage survey. On the 12th day after sowing, the number of normal germinated seeds (with the length of radicle extending out of the seed coat ≥ seed length and plumule length ≥ 50% seed length) was counted, and the germination rate (%) was calculated as (number of normal germinated seeds per hill/number of seeds per hill) × 100%. On the 20th day after sowing, the number of effective seedlings (seedling height ≥ 50% of the average) was investigated, and the seedling percentage (%) was calculated as (number of effective seedlings per hill/number of seeds per hill) × 100%. During the seedling percentage survey, 3 hills of rice with uniform growth were collected from each site to determine the plant height, foliar age, stem and leaf fresh weight, and stem and leaf dry weight.

#### 2.3.2 Determination of plant antioxidant enzyme activity and oxidative damage

Superoxide dismutase (SOD) activity was determined using the NBT photoreduction inhibition method (Giannopolitis and Ries, 1977), the catalase (CAT) activity was determined using the H_2_O_2_ degradation rate method (Maehly, 1954), the peroxidase (POD) activity was determined using the guaiacol oxidation method (Maehly, 1954), and the MDA content was determined by the thiobarbituric acid method (Jambunathan, 2010).

#### 2.3.3 Determination of dissolved oxygen content in the field

At 9:00 am every second day from the day of sowing, a JPB-607A portable dissolved oxygen meter was used to determine the dissolved oxygen content in water at the water-soil boundary in the plots with water direct seeding, and measurements were performed at 15 random sites in each plot.

#### 2.3.4 Variety evaluation and yield estimation

One day before harvest, 10 hills of rice plants were collected from each plot to determine the yield components and calculate the number of effective panicles, the number of grains per panicle, the number of unfilled grains, and the 1000-grain weight. After removing 2 rows at the boundaries of each plot, the rice was manually harvested, and the yield was calculated.

#### 2.3.5 Energy input, output, and use efficiency in rice production

Taking a demonstration area of 77.47 hm^2^ as the subject of study, the whole-rice-life-cycle input data of production materials (e.g., fertilizers, fuels, machinery, and electricity) and labor were investigated, and the energy utilization efficiency and economic benefits were calculated. Based on whether the energy was renewable, the input factors were divided into renewable energy and non-renewable energy, with the former including labor, irrigation water, and seeds, while the latter covered machinery, fuels, fertilizers, and agricultural chemicals. On this basis, the actual usage of each input factor was multiplied by the corresponding energy conversion coefficients in **Supplementary Table 1** and summed to obtain the total energy input. Among them, the energy inputs of various machines were calculated separately according to the formula listed in **Supplementary Table 1**, and the machinery energy input is expressed as (Equation 1):

(1) $$\text{Energy input from machinery }\left(\text{EIM};{\text{MJnhm}}^{-2}\right)=\text{WHF}\times \text{MTR}\times \text{WOM}/\text{TOL}/\text{FEW}$$

where EIM, WHF, MTR, WOM, TOL, and FEW represent the energy input from machinery, the working hours in the field, the energy used to manufacture, transport, and repair the machinery, the weight of the machinery, the total overall life of the machinery, and field working efficiency, respectively.

The total energy output was obtained by summing the multiplications of the rice yield and the straw yield by the corresponding energy conversion coefficients.

Based on the energy input and output, the net energy (NE), energy use efficiency (EUE), and energy productivity (EP) (Demircan et al., 2006) were calculated using (Equations 2–4):

(2) $$\text{NE }{(}^{\text{MJ}\cdot {\text{ha}}^{-1}})=\text{energy output}-\text{energy input}$$

(3) $$\text{EUE }\left(\text{MJ}\cdot {\text{MJ}}^{-1}\right)=\text{energy output}/\text{energy input}$$

(4) $$\text{EP}\left(\text{kg}\cdot {\text{MJ}}^{-1}\right)=\text{rice yield}/\text{energy input}$$

#### 2.3.6 Economic benefits

The economic benefits were calculated based on the inputs (production materials and labor) and outputs (rice and straw) in the demonstration area to calculate the net income and output - input ratio using (Equations 5, 6).

(5) Net benefit=total benefit-production cost

(6) Output-to-input ratio=Total benefit/production cost

The cost of each production material and the average rice price are shown in **Supplementary Table 3**.

### 2.4 Statistics

Microsoft Excel 2017 (Microsoft Corp., Redmond, WA, USA) and SPSS 20 (IBM Corp., Armonk, NY, USA) were used for statistical analysis, and Origin 9 (OriginLab Corp., Northampton, MA, USA) was used for figure plotting.

## 3 Results

### 3.1 Effects of coating on the germination rate and seedling percentage of hybrid rice with different direct seeding methods

The direct seeding method and coating treatment significantly affected germination rate, seedling percentage, plant height, fresh weight, and dry weight of rice (**Table 1**). Wet direct seeding performed significantly better than water direct seeding, especially in terms of germination rate and seedling percentage (P< 0.01). The variety-coating interactions and direct seeding-coating interactions significantly affected most growth indicators (**Table 2**). With wet direct seeding, the coating and no-coating treatments did not significantly affect seedling percentage for either the flood-resistant or flood-sensitive variety, indicating limited performance of coating in improving seedling percentage under no water stress. However, the coating treatment significantly increased the fresh weight and dry weight of the seedlings (P< 0.05), indicating its effectiveness in promoting seedling growth. With water direct seeding, the coating treatment significantly promoted the germination, seedling establishment, and growth of the two varieties. Specifically, the coating treatment increased the seedling percentage, plant height, fresh weight, and dry weight by 25.6%, 50.3%, 32.6%, and 165%, respectively, in the flood-resistant variety and by 78.5%, 50.6%, 91.3%, and 109.7%, respectively, in the flood-sensitive variety. The significantly increased seedling percentage of the flood-sensitive variety with oxygen-releasing coating suggested that the coating effectively alleviated the inhibited germination of the flood-sensitive variety under flooding conditions. Overall, coating significantly improved the germination and seedling growth of rice under flooding conditions, and the promotion effects on the flood-sensitive variety were generally stronger than on the flood-resistant variety.

**Table 1:** Variance analysis of the effects of coating on hybrid rice seedling growth with different direct seeding methods.

ANOVA	Germination rate	Seedling percentage	Seedling height	Leaf age	Fresh weight	Dry weight
V	192.40**	59.08*	10.85ns	49.69*	998.56**	135.56**
P	1555.46**	2638.58**	234.1**	2.54ns	3441.44**	750.2**
C	174.09**	173.13**	148.7**	12.87**	2748.18**	1905.05**
V×P	13.20ns	309.81**	48.3**	8.14*	317.54**	30.01**
V×C	34.39**	39.6**	6.48*	1.05	40.9**	1.05ns
P×C	77.37**	139.78**	18.16**	2.98	55.67**	4.69ns
V×P×C	95.52**	42.04**	5.36*	0.53	1010.18**	2.92ns

V, P, and C represent variety, direct seeding method, and coating treatment, respectively; * and ** represent significant differences at 0.05 and 0.01 levels, respectively; ns represents no significant difference. V × P, V × C, P × C, and V × P × C represent interactions among variety, direct seeding method, and coating.

**Table 2:** Effects of coating on hybrid rice seedling growth with different direct seeding methods.

Treatments			Germination rate (%)	Seeding percentage (%)	Seeding height (cm)	Leaf age	Fresh weight (g)	Dry weight (g)
V1	P1	C1	87.67a	79.67a	19.27a	2.72ab	3.63b	0.73b
		C2	82.00a	78.33a	15.29b	2.28c	2.20e	0.40e
	P2	C1	59.67b	52.33c	15.47b	2.48bc	2.44d	0.53d
		C2	55.33b	41.67d	10.29c	2.34c	1.84f	0.20g
V2	P1	C1	67.33b	60.67b	14.6b	2.89a	3.83a	0.93a
		C2	66.33b	60.33b	13.65b	2.67ab	3.72b	0.64c
	P2	C1	67.67b	58.33b	15.00b	2.85a	3.06c	0.65c
		C2	34.67e	32.67c	9.96c	2.75ab	1.60g	0.31f

V1: Jinyou 1319; V2: Jingliangyou 1377; P1: wet direct seeding; P2: water direct seeding; C1: coating; C2: no-coating; Different letters following the data in the same column indicate that the same variety shows significant differences at the 5% level under different treatments.

### 3.2 Effects of coating on the physiological characteristics of hybrid rice and the dissolved oxygen content under different direct seeding methods

**Figure 2:**
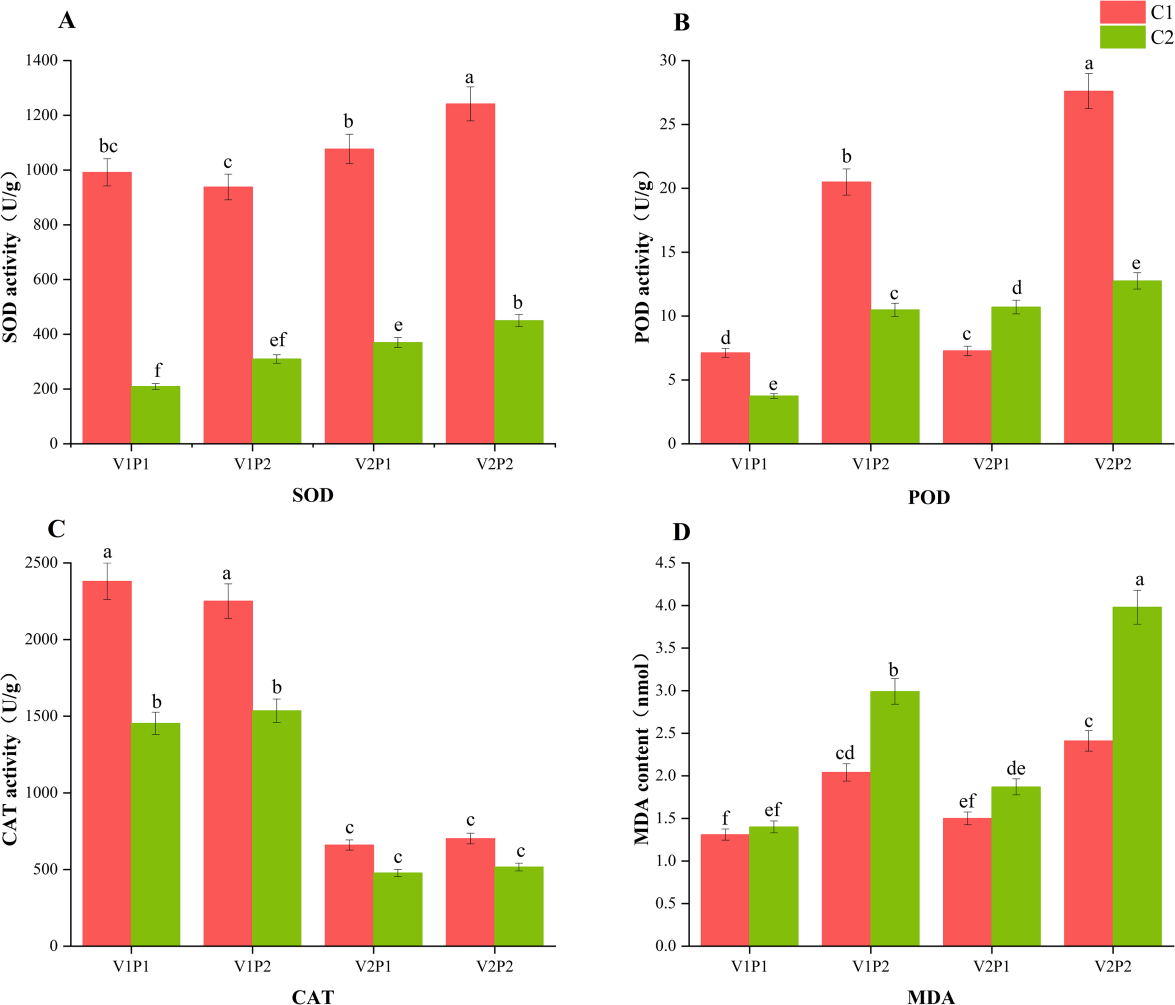
Antioxidant system activity and oxidative damage indicators. **(A)** SOD activity; **(B)** POD activity; **(C)** CAT activity; **(D)** MDA content. (Different letters marking the data indicate significance at the 5% level among the flooding treatments with the same variety).

As shown in **Figure 2**, variety and coating treatment significantly affect SOD, POD, and CAT activities and the MDA content of the rice seedlings. In addition, MDA was also affected by the interaction between water and coating. Coating significantly increased the activity of SOD, a primary antioxidant enzyme, with the flood-resistant variety showing greater increases. The POD and CAT activities showed similar trends, with the V1 variety showing more significant increases. The interactive effects of direct seeding methods and coating treatment manifested as follows: With the water direct seeding method, coating significantly reduced the MDA content relative to the no-coating treatment. With the wet direct seeding method, coating treatments caused no significant difference in the MDA content. Thus, coating can effectively enhance the antioxidant enzyme system and alleviate the oxidative damage caused by hypoxia stress in flooded environments.

**Figure 3:**
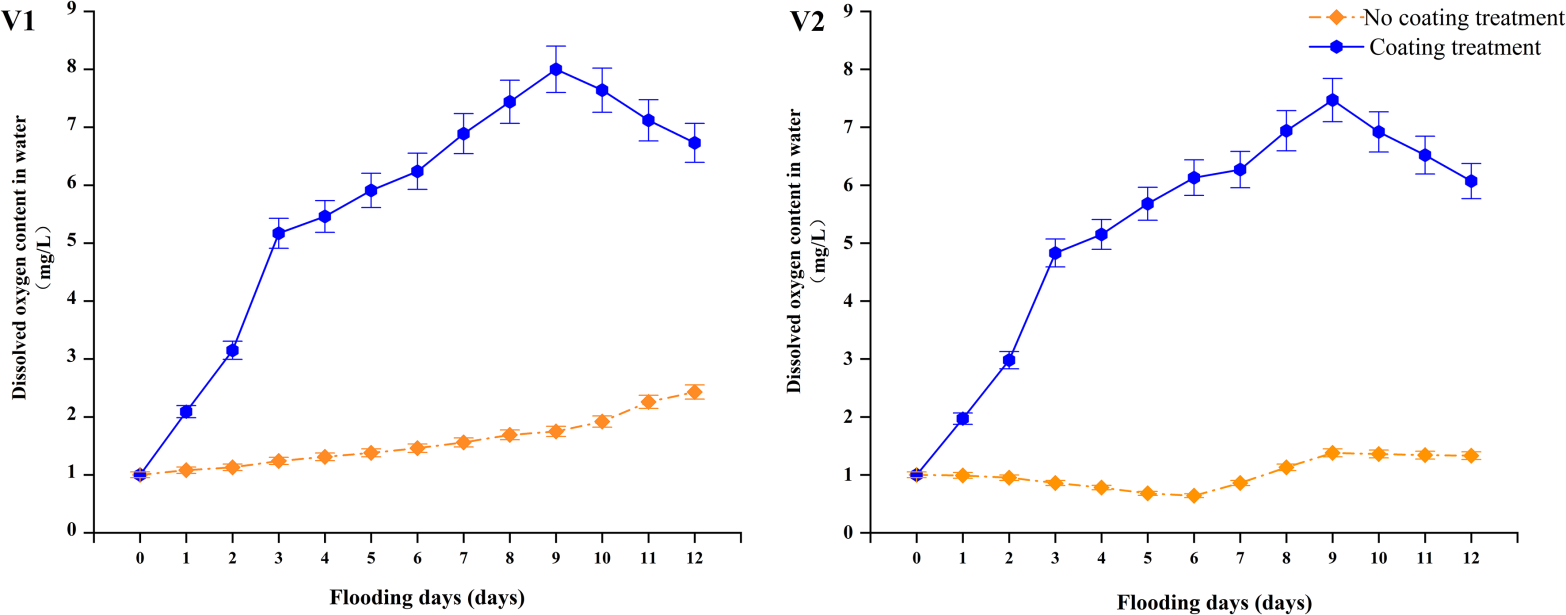
Dissolved oxygen content in water. V1: Jinyou 1319; V2: Jingliangyou 1377.

As shown in **Figure 3**, the oxygen-releasing coating significantly improved the dissolved oxygen content in the water layer of the rice field, increasing it to 4.53 times (on average) that without coating. Variety showed no direct effect on the dissolved oxygen content, with similar dissolved oxygen content curves between varieties. Specifically, a rapid increase was observed from 0 to 9 days (daily average increase of 0.7 to 0.8 mg·L^−1^), and peaks were observed on day 9 (V1: 8 mg·L^−1^; V2: 7.47 mg·L^−1^), reaching 4.57 times and 5.41 times that under the no-coating treatment. A period of decline spanned from day 9 to day 12, with decreases of 15.83% and 18.75%, respectively, and the dissolved oxygen content under the no-coating treatment slightly increased or remained stable. With the termination of the flooding treatment at day 12, the water layer’s dissolved oxygen content under the coating treatment still reached 3.67 times (on average) that of the no-coating treatment. Therefore, the oxygen-releasing coating agent can maintain a long-term stable oxygen supply.

### 3.3 Effects of coating on hybrid rice yield and yield components under different direct seeding methods

The average yield of the flood-resistant variety was significantly higher than that of the flood-sensitive variety, and the average yield with water direct seeding decreased by 1038.36 kg·hm^−2^ compared with wet direct seeding (**Table 3**). In this study, the yield was closely related to the number of effective panicles, while the number of effective panicles was significantly regulated by the direct seeding method (F = 48.09**), coating (F = 53.39**), and the interaction between the two (F = 20.06**) (**Table 4**). By significantly increasing the number of effective panicles and the seed setting rate, coating significantly improved rice yield under water direct seeding, achieving yield increases of 1319.93 kg·hm^−2^ and 1617.31 kg·hm^−2^ for the flood-resistant and flood-sensitive varieties, respectively. Using the seed coating, the yield with water direct seeding showed no significant difference compared to that under wet direct seeding and no-coating conditions, and the flood-sensitive variety even achieved higher yields instead.

**Table 3:** Effects of coating on hybrid rice yield and yield components with different direct seeding methods.

Treatments			Effective panicles (×10 ^4/^hm^−2^)	Grains per panicle (No.)	Seed-setting rate (%)	1000-grain weight (g)	Grain yield (kg hm^−2^)
V1	P1	C1	210.67a	199.11c	59.87b	31.05a	7682.55a
		C2	213.33a	206.99bc	56.62bc	30.81a	7385.94ab
	P2	C1	208.00a	206.67bc	58.41b	31.09a	7021.25bc
		C2	198.67b	210.90bc	50.77c	30.90a	5701.32e
V2	P1	C1	209.33a	209.31bc	66.97a	25.03b	6951.21cd
		C2	198.67b	244.86a	55.29bc	25.14b	6654.54d
	P2	C1	209.33a	224.99ab	60.70b	25.23b	6707.78cd
		C2	185.33c	217.55bc	52.04c	25.20b	5090.47f

V1: Jinyou 1319; V2: Jingliangyou 1377; P1: wet direct seeding; P2: water direct seeding; C1: coating; C2: no-coating; Different letters following the data in the same column indicate that the same variety shows significant differences at the 5% level under different treatments.

**Table 4:** Variance analysis of the effects of coating on hybrid rice yield and yield components with different direct seeding methods.

ANOVA	Effective panicles	Grains per panicle	Seed-setting rate	1000-grain weight	Grain yield
V	147.00**	49.98*	6.44	2689.18**	67.06*
P	48.09**	0.01	15.27*	0.35	319.19**
C	53.39**	5.64*	46.06**	0.21	428.45**
V×P	0.82	2.15	0.26	0.04	5.36
V×C	24.50**	0.89	4.21	0.43	3.04
P×C	20.06**	7.59*	0.9	0.01	188.86**
V×P×C	0.06	5.40*	2.6	0.06	3.04

V, P, and C represent variety, direct seeding method, and coating treatment, respectively; * and ** represent significant differences at 0.05 and 0.01 levels, respectively; ns represents no significant difference. V × P, V × C, P × C, and V × P × C represent interactions among variety, direct seeding method, and coating.

### 3.4 Effects of coating on the energy utilization of hybrid rice with different direct seeding methods

#### 3.4.1 Effects of coating on energy input of hybrid rice with different direct seeding methods

**Figure 4:**
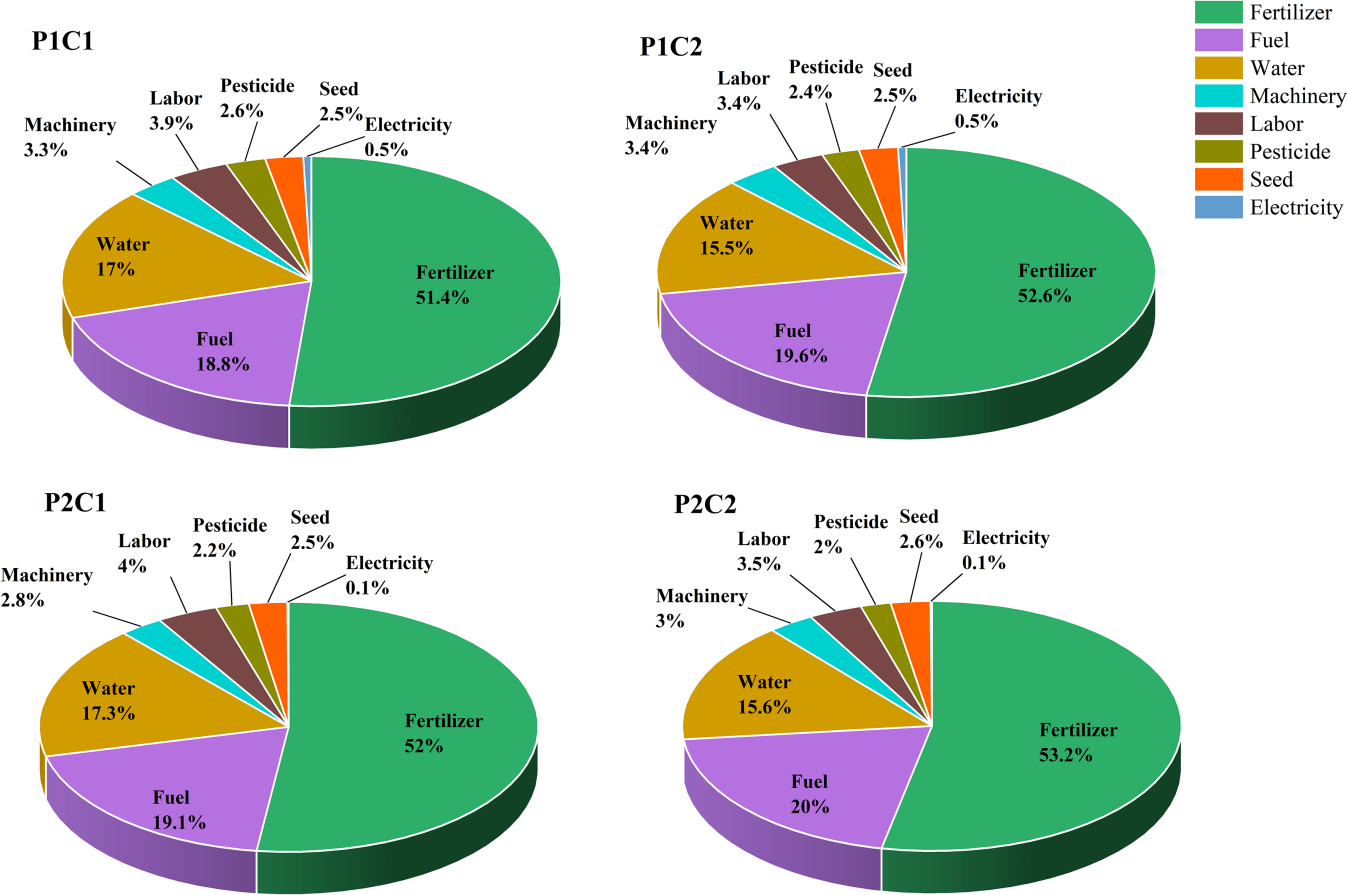
Effects of coating on the energy input of hybrid rice with different direct seeding methods.

As shown in **Table 5**, rice varieties, direct seeding methods, and coating treatments have little impact on the energy input. Specifically, the coating treatment increased energy input of the V1 variety by only 1.42% and 0.96%, respectively, compared to the no-coating treatment under water direct seeding and wet direct seeding conditions. Such increases in the V2 variety reached 0.79% and 1.89%, respectively. Thus, the coating did not significantly increase the energy input. The energy inputs for different production materials ranked from highest to lowest were: fertilizers > fuels > irrigation water > labor > machinery > seeds > pesticides > electricity (**Figure 4**). Together accounting for > 70% of the total energy input, those of fertilizers and fuels showed small differences between different direct seeding methods and coating treatments. Although water direct seeding did not significantly reduce the energy input of the production system, it required no drainage during sowing, thus reducing the frequency of subsequent drainage and irrigation. As a result, the labor and irrigation water inputs were reduced. In addition, flooding direct seeding eliminated the need for weed control by suppressing weeds with water, effectively reducing the use of chemical herbicides. These three improvements are crucial for the green and sustainable development of rice production in China.

**Table 5:** Effects of coating on energy input of hybrid rice with different direct seeding methods.

Items	V1				V2			
	P1		P2		P1		P2	
	C1	C2	C1	C2	C1	C2	C1	C2
Ploughing machine	437.65	446.42	453.95	461.47	442.66	437.65	448.93	453.95
Coating machine	119.9	0	119.6	0	119.72	0	120.02	0
Sprayer	1.7	1.73	1.67	1.73	1.73	1.71	1.64	1.7
Harvester	225.05	226.17	228.41	226.17	227.08	225.05	222.81	227.29
Labor	921.65	932.49	794.68	800.13	917.81	925.86	788.63	799.41
Fuel	4472.7	4534.64	4598.28	4625.3	4521.13	4472.7	4522.26	4589.27
Electricity	126.76	20.02	126.43	19.66	127.15	19.8	126.5	20.05
N fertilizer	9689.51	9704.06	9694.8	9653.13	9708.03	9642.55	9595.59	9664.38
P fertilizer	919.56	916.58	907.62	914.71	911.6	914.22	908	915.21
K fertilizer	1663.25	1657.23	1663.58	1658.34	1658.67	1661.91	1660.57	1664.14
Water	4057.18	4076.67	3588.53	3593.73	4067.72	4072.16	3601.28	3600.02
Pesticide	211.51	214.54	212.52	209.48	209.48	210.5	211.51	206.45
Herbicide	168.98	164.22	109.48	111.86	164.22	157.08	114.24	109.48
Fungicide	239.76	131.76	235.44	129.6	241.92	133.92	244.08	131.76
Seeds	585.5	587.56	584.03	588.29	584.77	586.38	586.09	586.82
Total energy	23840.66	23614.09	23319.03	22993.61	23903.91	23461.68	23152.15	22969.92

V1: Jinyou 1319; V2: Jingliangyou 1377; P1: wet direct seeding; P2: water direct seeding; C1: coating; C2: no-coating.

#### 3.4.2 Effect of coating on energy use efficiency of hybrid rice with different direct seeding methods

Analysis of variance in **Table 6** shows that coating has extremely significant effects on energy output, net energy, energy use efficiency, and energy productivity, and that the direct seeding method-coating treatment interactions show extremely significant effects on those indicators. As shown in **Table 7**, the overall energy output of the flood-resistant variety is 13.19% higher than that of the flood-sensitive variety, and the average energy output with water direct seeding is 15.63% lower than that with wet direct seeding. Under the flooded conditions, coating increased the energy output of V1 and V2 by 14.26% and 22.84%, respectively. The NE, EUE, and EP of V1 with coating improved by 16.2%, 12.7%, and 21.5%, respectively, compared with the no-coating treatment, while the improvements of V2 were even larger (26.8%, 21.9%, and 30.7%), suggesting that coating is more effective for the flood-sensitive variety with water direct seeding.

**Table 6:** Variance analysis of the effects of coating on the energy use efficiency of hybrid rice with different direct seeding methods.

ANOVA	Energy output	NE	EUE	EP
V	336.04**	334.08**	304.63**	62.30*
P	488.83**	467.97**	336.08**	226.61**
C	255.54**	245.50**	191.07**	355.66**
V×P	27.41**	27.65**	26.67**	5.43ns
V×C	3.10ns	3.02ns	3.26ns	2.92ns
P×C	251.42**	252.93**	280.67**	205.66**
V×P×C	12.45**	13.15**	19.32**	5.63*

V, P, and C represent variety, direct seeding method, and coating treatment, respectively; * and ** represent significant differences at 0.05 and 0.01 levels, respectively; ns represents no significant difference. V × P, V × C, P × C, and V × P × C represent interactions among variety, direct seeding method, and coating.

**Table 7:** Effects of coating on the energy use efficiency of hybrid rice with different direct seeding methods.

Treatments			Energy output (MJ·hm^−2^)	NE (MJ·hm^−2^)	EUE (MJ·MJ^−1^)	EP (Kg·MJ^−1^)
V1	P1	C1	220926.65a	197085.98a	9.27a	0.32a
		C2	219161.02a	195546.93a	9.28a	0.31ab
	P2	C1	198141.27b	174822.25b	8.50b	0.30bc
		C2	173409.13d	150410.51d	7.54d	0.25e
V2	P1	C1	189082.23c	165168.32c	7.91c	0.29cd
		C2	190607.23c	167145.76c	8.12c	0.28d
	P2	C1	185990.61c	162838.46c	8.03c	0.29cd
		C2	151404.61e	128434.69e	6.59e	0.22f

V1: Jinyou 1319; V2: Jingliangyou 1377; P1: wet direct seeding; P2: water direct seeding; C1: coating; C2: no-coating; Different letters following the data in the same column indicate that the same variety shows significant differences at the 5% level under different treatments.

### 3.5 Effects of coating on the economic benefits of hybrid rice with different direct seeding methods

**Figure 5:**
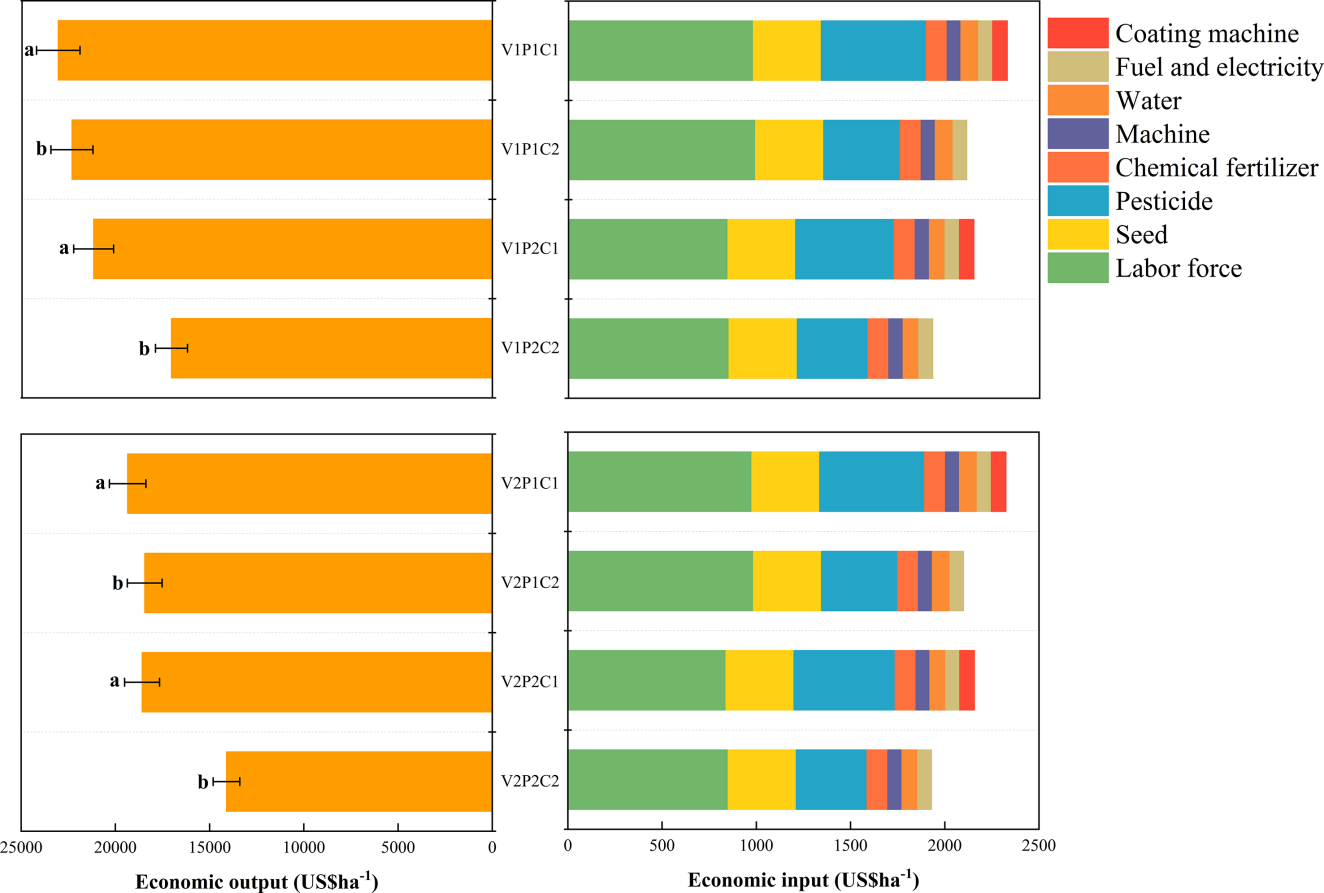
Effects of seed coating on the total economic cost, net income, and output-input ratio of hybrid rice under different direct seeding methods. (Different letters marking the data indicate significance at the 5% level among the flooding treatments with the same variety.).

As shown in **Figure 5**, water direct seeding significantly reduces the economic costs compared to wet direct seeding, mainly due to the reduced input in areas such as labor and irrigation water. Coating increased the consumption of machinery, labor, and coating agents, leading to significantly higher production costs than the no-coating treatment. With water direct seeding, coating increased the net profits of V1 and V2 by 281.73 USD·hm^−2^ and 387.18 USD·hm^−2^, respectively, and their output-to-input ratios by 11.61% and 17.83%, respectively, mainly by significantly increasing yield. In contrast, the coating treatment under wet direct seeding resulted in no significant improvement. In summary, the coating agent can optimize the economic benefits and resource utilization efficiency under the water direct seeding mode, especially for water-sensitive varieties.

## 4 Discussion

This study shows that the interactions of oxygen-releasing coating agent with water and rice variety significantly affect rice seed germination and seedling establishment, consistent with the conclusions of Javed et al. (2021) and Zhang et al. (2023). Although previous studies have confirmed the technical advantages of oxygen-releasing coating (Dueñas et al., 2024), the coating agent developed by our research team showed high efficiency in alleviating hypoxia stress in this study. The coating treatment significantly increased the seedling percentage of the flood-sensitive variety from 32.67% to 60.67%, highlighting its potential application values in actual production. With continuous oxygen release from the reaction between calcium peroxide and water, the coating significantly increased the dissolved oxygen content in the water layer, creating a beneficial oxygen-rich microenvironment for the rice root system (Hu et al., 2020). Consequently, the rice antioxidant defense system was activated: First, the SOD activity was significantly increased (Yuan et al., 2020). As the core initialization process of the antioxidant response, the increased SOD activity triggers a cascade reaction that stimulates synergistic increases in POD and CAT activities. Ultimately, the excess active oxygen accumulated under hypoxic stress was effectively eliminated, and lipid peroxidation damage was reduced, manifesting as a significant decrease in the MDA content (Du et al., 2025). From the morphogenesis perspective, the coating agent significantly increases the seedling percentage by improving the oxygen in the environment, and from the physiological perspective, it systematically enhances the resistance of rice seedlings to hypoxic stress (Zhang et al., 2018). This finding complements previous studies that focused primarily on seedling percentage, revealing potential pathways through which coating affects yield by promoting early growth (Biswas et al., 2001). In terms of research presentation, this study has not incorporated imaging evidence to assist in the demonstration of coating evenness and seedling phenotype differences. Future research may employ imaging methods, such as SEM, to better characterize the coating and the seedling status under different treatments, thereby advancing our understanding of the mechanisms underlying the functioning of the oxygen-releasing coating.

In this study, the oxygen-releasing coating improved rice yield mainly by increasing the number of effective panicles, with a contribution rate significantly higher than that of the number of grains per panicle, the seed setting rate, and the 1000-grain weight, aligning with the conclusion of Xiong et al. (2005). Through continuous oxygen supply, the coating significantly improves the seed germination and seedling establishment under flooding stress, providing the necessary seedling quantities for the construction of high-yield populations (Javed et al., 2021). Specifically, the number of effective panicles of the flood-resistant variety (V1) and the flood-sensitive variety (V2) increased by 4.69% and 12.95%, respectively, effectively offsetting the yield loss caused by flooding. Thus, the yield with water direct seeding became comparable to that of conventional wet direct seeding. This effect is particularly applicable to uneven rice fields in hilly regions. With seed coating, rice plants in low-lying areas with water accumulation form populations with yield potential comparable to that in flat wet areas, providing an effective solution for the safe production of water-sensitive varieties under flooding conditions. This technology breaks through the long-standing yield bottleneck in direct-seeded rice under flooding stress, offering important technical support for its large-scale, simplified applications.

The optimized water resource management and weed control under the water direct seeding treatment significantly reduce labor and resource inputs during production. With the same seed coating treatment, water direct seeding reduces labor input by 13.7% to 14.1% compared to wet direct seeding, mainly due to the less frequent irrigation and agricultural chemical spraying operations. Meanwhile, its water layer significantly inhibits weed growth, reducing herbicide use and avoiding the environmental impact from agricultural chemicals. Although the coating treatment slightly increased the energy consumption at the seed treatment stage (Yang et al., 2014), the overall energy use efficiency of water direct seeding increased by 12% to 22%, reflecting its energy efficiency advantages. Unlike the serious imbalance in energy efficiency caused by weed hazards reported by Chauhan et al. (2015), the water direct seeding with coating treatment (V1P2C1) reached a total energy input (23,319.03 MJ·hm^−2^) 1.25% lower than that of the wet direct seeding with no-coating treatment (23,614.09 MJ·hm^−2^), indicating its better energy management performance. In particular, coating treatments (V1 and V2) with water direct seeding increased the energy output by 14.26% and 22.84%, respectively, compared with the no-coating treatments, much higher than the coating-induced increments (< 8%) observed in upland rice production in the Philippines, as reported by Quilty et al. (2014), highlighting the significant efficiency improvement potential of the coating technology in flooded environments.

The physical oxygenation mechanism of the coating activates the seed’s photosynthetic potential that is inhibited by hypoxia in flooded, hypoxic environments, and this activation is difficult to achieve under dry direct seeding conditions. This also explains the insignificant improvement in energy output by coating under wet direct seeding. Thus, the water direct seeding with coating treatment demonstrates the synergy of resource conservation, pollution prevention, and energy efficiency improvement. In terms of resources, this model significantly reduces irrigation water demand, making it especially suitable for water-scarce regions, and mitigates the risk of soil degradation through simplified lightweight production (Tabbal et al., 2002; Gupta et al., 2003). In the environmental aspect, it reduces nutrient loss and non-point source pollution caused by runoff and decreases environmental impact by lowering chemical use by 33% (Baruah and Dutta, 2007; Bhatt et al., 2016). In terms of energy efficiency, the energy productivity of water direct seeding with coating treatment (0.30 kg·MJ^−1^) is 20% higher than that of the water direct seeding without coating treatment (0.25 kg·MJ^−1^). Meanwhile, its energy use efficiency remains stable at a high range (6.59 to 8.50 MJ·MJ^−1^), outperforming that in water-rich regions (e.g., the typical value in Malaysia is 7.76 MJ·MJ^−1^) (Muazu et al., 2015). Thus, this model is particularly suitable for sustainable rice production under water constraints.

Economic costs and benefits are critical factors determining the adoption of new agricultural technologies. Although the coating increased the production cost by 221.67 USD compared to the no-coating production cost, it increased the output by 557.61 USD on average under water direct seeding conditions. In particular, the net gain with the flood-sensitive variety was about 21 times higher, significantly higher than the benefits of the water-saving technology reported by Lampayan et al. (2015) (usually< 20%). By achieving a higher yield (387.18 USD·hm^−2^) with a low incremental cost (227.53 USD·hm^−2^), this model provides economic viability for small-scale farmers managing uneven rice fields in hilly regions (Lu et al., 2018). Water direct seeding eliminated extensive soil preparation and overcame topographical constraints, while the coating compensated for yield loss under flooding stress (25% increase in yield in the flood-sensitive variety), thereby achieving optimal utilization of low-quality lands (Huang, 2018). Moreover, its relatively simple operations promise broad application potential in the context of China’s current rice production challenges, such as low profits, labor shortages, and limited education levels of farmers.

## 5 Conclusion

Water direct seeding (P2) with the oxygen-releasing coating significantly improved the seedling establishment and yield performance of the flood-sensitive variety (V2). With the coating, the seedling percentage increased significantly by 25.66 percentage points, and the seedling fresh weight and dry weight increased by 91.25% and 109.68%, respectively. Thus, the coating effectively promoted the early biomass accumulation while mitigating irregular seeding emergence and hindered growth due to flooding. The seed coating with water direct seeding model increased the number of effective panicles by 12.95% and the yield by 31.77%, basically matching the yield under wet direct seeding (P1). In terms of energy utilization, the coating treatment achieved significant (> 20%) improvements in net energy output, energy use efficiency, and energy productivity. Thus, it significantly improved the economic benefits and input-output ratio, allowing the flood-sensitive variety with water direct seeding to achieve better economic returns than under wet direct seeding. Although the model requires greater investment in coating equipment and imposes higher process requirements, it can achieve synergistic improvements in yield, efficiency, and energy conservation. Hence, it provides an integrated solution for lightweight, simplified rice production, which is especially suitable for application and generalization in hilly rice-planting regions with uneven fields and complex water resources management.

## Data Availability

The original contributions presented in the study are included in the article/**Supplementary Material**. Further inquiries can be directed to the corresponding authors.
