# Supplementary material for: Oxygen-releasing seed coating enhances yield and resource use efficiency in direct-seeded rice
Source: Front Plant Sci. 2026 Jan 27;17:1746831. doi: 10.3389/fpls.2026.1746831 (PMC12885989; doi:10.3389/fpls.2026.1746831)
Supplement: Supplementary file 1 [file DataSheet1.docx]

**Supplementary Information:**

**Supplementary Table S1. Energy equivalent of inputs and outputs in agricultural production.**

| Items | Unit | Energy equivalent (MJ unit^−1^) | References: |
| --- | --- | --- | --- |
| Input |  |  |  |
| 1. Machinery | h | 62.70 | (Erdal et al., 2007) (Mohammadi et al., 2008) |
| 2. Labor | h | 1.96 | (Yilmaz et al., 2005) |
| 3. Fuel | L | 56.31 | (Erdal et al., 2007) (Sayin et al., 2005) |
| 4. Electricity | kWh | 3.60 | ( Sahr et al., 2005) |
| 5. Fertilizer |  |  |  |
| N fertilizer | kg | 66.14 | (Esengun et al., 2007) |
| P fertilizer | kg | 12.44 | (Esengun et al., 2007) |
| K fertilizer | kg | 11.15 | (Karkacier and Gokalp Goktolga, 2005) |
| 6. Water | m^3^ | 1.02 | (Sayin et al., 2005) (Acaroğlu and Şemi Aksoy, 2005) |
| 7. Agricultural chemicals |  |  |  |
| Pesticide | kg | 101.20 | (Ozkan et al., 2007) |
| Herbicide | kg | 238.00 | (Ozkan et al., 2007) |
| Fungicide | kg | 216.00 | (Pathak and Bining, 1985) |
| 8. Seeds | kg | 14.70 | (Ozkan et al., 2004) |
| Output |  |  |  |
| 1. Rice grain | kg | 17.00 | (Ozkan et al., 2004) |
| 2. Rice straw | kg | 12.50 | (Ozkan et al., 2004) |

**Supplementary Table S2. Parameters required for estimating machinery energy inputs.**

| Machinery | Weight (kg) | Fuel/power consumption  (L h^−1^ or W ) | Operational life (h) | Field working efficiency (%) | Power source |
| --- | --- | --- | --- | --- | --- |
| Ploughing machine | 3250.00 | 13.40 | 2500.00 | 65.00 | Fuel oil |
| Coating machine | 108.00 | 1500.00 | 1500.00 | 75.00 | Electricity |
| Agricultural chemical sprayer | 42.00 | 4600.00 | 1000.00 | 85.00 | Electricity |
| Harvester | 3750.00 | 16.25 | 3500.00 | 60.00 | Fuel oil |

**Supplementary Table 3. Unit prices of rice and inputs for economic benefit evaluation.**

| Items | Unit | Price (USD) |
| --- | --- | --- |
| Ploughing machine, harvester | h | 13.30 |
| Coating machine | h | 3.98 |
| Agricultural chemical sprayer | h | 1.44 |
| Labor | h | 2.11 |
| Fuel | L | 0.91 |
| N fertilizer | kg | 0.28 |
| P fertilizer | kg | 0.10 |
| K fertilizer | kg | 0.43 |
| Water | m^3^ | 0.02 |
| Pesticide | kg | 69.40 |
| Herbicide | kg | 115.67 |
| Fungicide | kg | 303.64 |
| Seeds | kg | 9.17 |
| Electricity | kWh | 0.08 |
| Rice grain | kg | 0.38 |
| Rice straw | kg | 0.0042 |

**Supplementary Table 4. Effects of coating on the inputs of various elements of hybrid rice with different direct seeding methods.**

| Items | | V1 | | | | V2 | | | |
| --- | --- | --- | --- | --- | --- | --- | --- | --- | --- |
|  |  | P1 | | P2 | | P1 | | P2 | |
| Items | Units | C1 | C2 | C1 | C2 | C1 | C2 | C1 | C2 |
| Ploughing machine | h∙hm^-2^ | 3.49 | 3.56 | 3.62 | 3.68 | 3.53 | 3.49 | 3.58 | 3.62 |
| Coating machine | h∙hm^-2^ | 19.92 | 0 | 19.87 | 0 | 19.89 | 0 | 19.94 | 0 |
| Agricultural chemical sprayer | h∙hm^-2^ | 0.55 | 0.56 | 0.54 | 0.56 | 0.56 | 0.55 | 0.53 | 0.55 |
| Harvester | h∙hm^-2^ | 2.01 | 2.02 | 2.04 | 2.02 | 2.03 | 2.01 | 1.99 | 2.03 |
| Labor | man-h∙hm^-2^ | 470.23 | 475.76 | 405.45 | 408.23 | 468.27 | 472.38 | 402.36 | 407.86 |
| Fuel | L∙hm^-2^ | 79.43 | 80.53 | 81.66 | 82.14 | 80.29 | 79.43 | 80.31 | 81.5 |
| Electricity | kWh∙hm^-2^ | 35.21 | 5.56 | 35.12 | 5.46 | 35.32 | 5.5 | 35.14 | 5.57 |
| N fertilizer | kg∙hm^-2^ | 146.5 | 146.72 | 146.58 | 145.95 | 146.78 | 145.79 | 145.08 | 146.12 |
| P fertilizer | kg∙hm^-2^ | 73.92 | 73.68 | 72.96 | 73.53 | 73.28 | 73.49 | 72.99 | 73.57 |
| K fertilizer | kg∙hm^-2^ | 149.17 | 148.63 | 149.2 | 148.73 | 148.76 | 149.05 | 148.93 | 149.25 |
| Water | m^3^∙hm^-2^ | 3977.63 | 3996.73 | 3518.17 | 3523.26 | 3978.96 | 3992.31 | 3530.67 | 3529.43 |
| Pesticide | kg∙hm^-2^ | 2.09 | 2.12 | 2.1 | 2.07 | 2.07 | 2.08 | 2.09 | 2.04 |
| Herbicide | kg∙hm^-2^ | 0.71 | 0.69 | 0.46 | 0.47 | 0.69 | 0.66 | 0.48 | 0.46 |
| Fungicide | kg∙hm^-2^ | 1.11 | 0.61 | 1.09 | 0.6 | 1.12 | 0.62 | 1.13 | 0.61 |
| Seeds | kg∙hm^-2^ | 39.83 | 39.97 | 39.73 | 40.02 | 39.78. | 39.89 | 39.87 | 39.92 |

Note: V1: Jinyou 1319; V2: Jingliangyou 1377; P1: wet direct seeding; P2: flooded direct seeding; C1: coating; C2: no coating.

**References:**

Acaroğlu, M., and Şemi Aksoy, A. 2005. The cultivation and energy balance of Miscanthus×giganteus production in Turkey. *Biomass Bioenergy* ,29, 42–48. doi: 10.1016/j.biombioe.2005.01.002

Bockari-Gevao, Sahr & Wan Ismail, Wan Ishak & Yahya, Azmi & Wan, Chan. 2005. Analysis of energy consumption in lowland rice-based cropping system of Malaysia. *Songklanakarin Journal of Science and Technology*. 27. 820-827.

Erdal, G., Esengün, K., Erdal, H., and Gündüz, O. 2007. Energy use and economical analysis of sugar beet production in Tokat province of Turkey. *Energy* ,32, 35–41. doi: 10.1016/j.energy.2006.01.007

Esengun, K., Gündüz, O., and Erdal, G. 2007. Input–output energy analysis in dry apricot production of Turkey. Energy Convers. Manag. 48, 592–598. doi:10.1016/j.enconman.2006.06.006

Karkacier, O, and Gokalp Goktolga, Z.2005. Input–output analysis of energy use in agriculture. *Energy Convers Management*. 46, 1513–1521. doi: 10.1016/j.enconman.2004.07.011

Mohammadi, A., Tabatabaeefar, A., Shahin, S., Rafiee, S., and Keyhani, A. 2008. Energy use and economical analysis of potato production in Iran a case study: Ardabil province. *Energy Convers Management*. 49, 3566–3570. doi: 10.1016/j.enconman.2008.07.003

Ozkan, B., Akcaoz, H., and Fert, C. 2004. Energy input–output analysis in Turkish agriculture. Renew. *Energy,* 29, 39–51. doi: 10.1016/S0960-1481(03)00135-6

Ozkan, B., Fert, C., and Karadeniz, C. F. 2007. Energy and cost analysis for greenhouse and open-field grape production. *Energy,* 32, 1500–1504. doi: 10.1016/j.energy.2006.09.010

Pathak, B. S., and Bining, A. S. 1985. Energy use pattern and potential for energy saving in rice-wheat cultivation. *Energy Agric*. 4, 271–278. doi: 10.1016/0167-5826(85)90022-1

Sayin, C., Nisa Mencet, M., and Ozkan, B. 2005. Assessing of energy policies based on Turkish agriculture:: current status and some implications. *Energy Policy,* 33, 2361–2373. doi: 10.1016/j.enpol.2004.05.005

Yilmaz, I., Akcaoz, H., and Ozkan, B. 2005. An analysis of energy use and input costs for cotton production in Turkey. Renew. *Energy* 30, 145–155. doi: 10.1016/j.renene.2004.06.001
